# Unveiling the therapeutic potential of Dl-3-n-butylphthalide in NTG-induced migraine mouse: activating the Nrf2 pathway to alleviate oxidative stress and neuroinflammation

**DOI:** 10.1186/s10194-024-01750-1

**Published:** 2024-04-02

**Authors:** Yingyuan Liu, Zihua Gong, Deqi Zhai, Chunxiao Yang, Guangshuang Lu, Shuqing Wang, Shaobo Xiao, Chenhao Li, Ludan Chen, Xiaoxue Lin, Shuhua Zhang, Shengyuan Yu, Zhao Dong

**Affiliations:** 1 Department of Neurology, the First Medical Center, Chinese PLA General Hospital, Fuxing Road 28, Haidian District, Beijing, 100853 China; 2 Medical School of Chinese PLA, Beijing, 100853 China; 3 School of Medicine, Nankai University, Tianjin, 300071 China; 4 Clinical School of Anhui Medical University, The Third Medical Center of Chinese PLA General Hospital, Beijing, 100039 China; 5 Department of Neurology, Bethune International Peace Hospital, Shijiazhuang, Hebei 050082, Hebei, China

**Keywords:** NBP, Nrf2, Neuroinflammation, Oxidative stress, Central sensitization, Migraine

## Abstract

**Background:**

Migraine stands as a prevalent primary headache disorder, with prior research highlighting the significant involvement of oxidative stress and inflammatory pathways in its pathogenesis and chronicity. Existing evidence indicates the capacity of Dl-3-n-butylphthalide (NBP) to mitigate oxidative stress and inflammation, thereby conferring neuroprotective benefits in many central nervous system diseases. However, the specific therapeutic implications of NBP in the context of migraine remain to be elucidated.

**Methods:**

We established a C57BL/6 mouse model of chronic migraine (CM) using recurrent intraperitoneal injections of nitroglycerin (NTG, 10 mg/kg), and prophylactic treatment was simulated by administering NBP (30 mg/kg, 60 mg/kg, 120 mg/kg) by gavage prior to each NTG injection. Mechanical threshold was assessed using von Frey fibers, and photophobia and anxious behaviours were assessed using a light/dark box and elevated plus maze. Expression of c-Fos, calcitonin gene-related peptide (CGRP), Nucleus factor erythroid 2-related factor 2 (Nrf2) and related pathway proteins in the spinal trigeminal nucleus caudalis (SP5C) were detected by Western blotting (WB) or immunofluorescence (IF). The expression of IL-1β, IL-6, TNF-α, Superoxide dismutase (SOD) and malondialdehyde (MDA) in SP5C and CGRP in plasma were detected by ELISA. A reactive oxygen species (ROS) probe was used to detect the expression of ROS in the SP5C.

**Results:**

At the end of the modelling period, chronic migraine mice showed significantly reduced mechanical nociceptive thresholds, as well as photophobic and anxious behaviours. Pretreatment with NBP attenuated nociceptive sensitization, photophobia, and anxiety in the model mice, reduced expression levels of c-Fos and CGRP in the SP5C and activated Nrf2 and its downstream proteins HO-1 and NQO-1. By measuring the associated cytokines, we also found that NBP reduced levels of oxidative stress and inflammation. Most importantly, the therapeutic effect of NBP was significantly reduced after the administration of ML385 to inhibit Nrf2.

**Conclusions:**

Our data suggest that NBP may alleviate migraine by activating the Nrf2 pathway to reduce oxidative stress and inflammation in migraine mouse models, confirming that it may be a potential drug for the treatment of migraine.

**Graphical Abstract:**

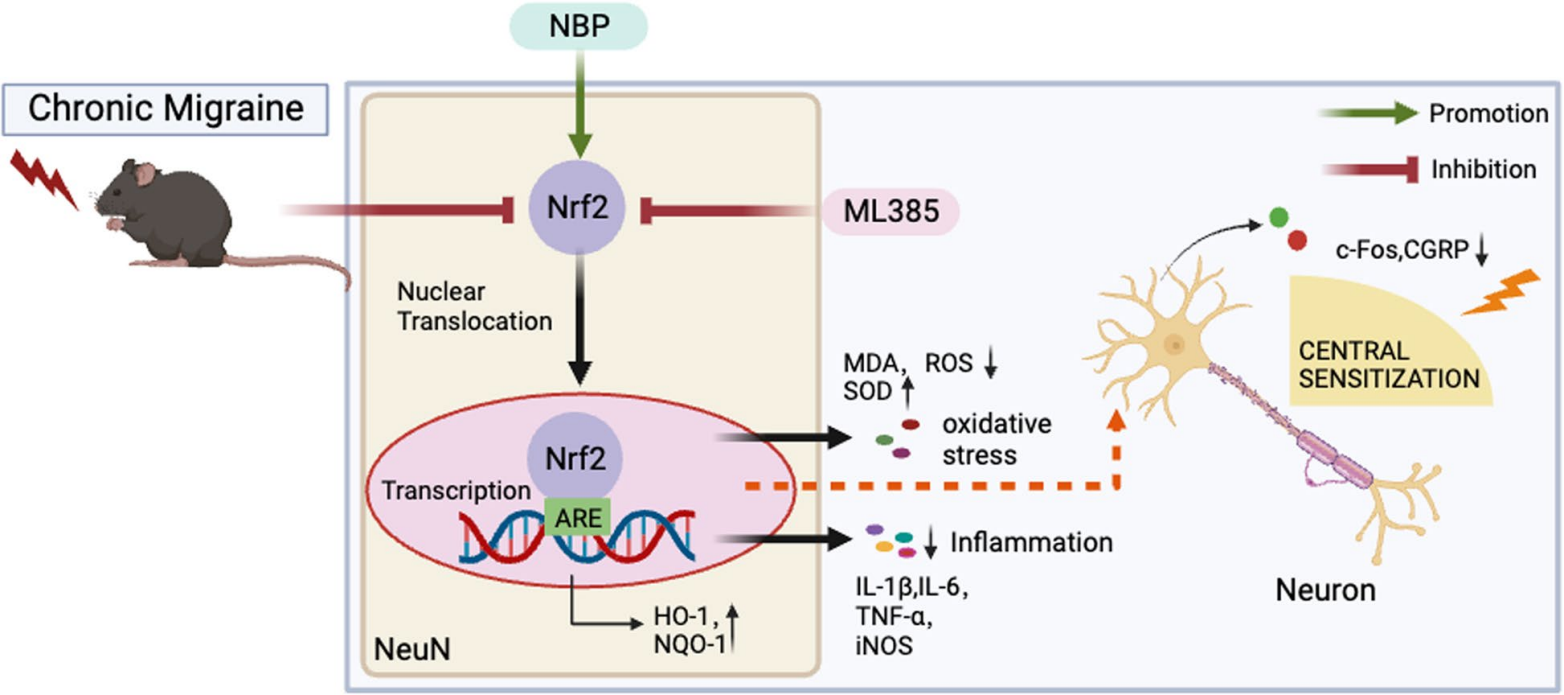

**Supplementary Information:**

The online version contains supplementary material available at 10.1186/s10194-024-01750-1.

## Introduction

Chronic migraine represents a debilitating neurological condition affecting approximately 2% of the general population. Individuals afflicted by chronic migraines endure headaches at least 15 days per month, accompanied by visual, auditory, and olfactory disturbances, as well as symptoms like nausea and vomiting. This condition imposes a significant burden on affected individuals, their families, and society at large [1]. Moreover, chronic migraine often co-occurs with sleep disturbances, anxiety, and depression, compounding the overall health challenges faced by sufferers [2]. Existing international guidelines for migraine treatment include acute analgesic treatment and preventive treatment to reduce the frequency of headache attacks [3].

However, commonly prescribed preventive medications are known to have varying degrees of adverse effects. For instance, amitriptyline has potential adverse effects such as heart failure, contraindications with monoamine oxidase inhibitors and selective 5-hydroxytryptamine reuptake inhibitors, and an increased risk of glaucoma [4]. Flunarizine may lead to adverse effects like Parkinson’s disease and depression [5]. Valproate has potential adverse effects including liver disease, thrombocytopenia, and negative impacts on female fertility [2, 6, 7]. Novel CGRP or CGRP receptor blockade has demonstrated the effectiveness in migraine patients [8, 9]. Nonetheless, the use of monoclonal antibodies has limitations, including high cost and potential side effects like rash, itching, and pain [6]. As a result, there is an urgent need to discover a more affordable, safe, and efficacious medication for preventing migraine attacks.

Currently, the precise mechanism underlying migraine remains elusive. Recently, an increasing number of studies have shown that oxidative stress (OS) and neuroinflammation play a key role in the pathogenesis of migraine [10, 11]. Oxidative stress, which results from an imbalance between the production of reactive oxygen species (ROS) and the elimination of antioxidant defense mechanisms, is associated with various headache disorders, including migraine [12–16], and endogenous ROS can cause oxidative damage to DNA, lipids, and proteins [17]. In contrast, reactive oxygen scavengers attenuated nociceptive hypersensitivity in rats with neuropathic pain, suggesting a possible role for antioxidants [18, 19]. Additionally, the role of inflammation in migraine is increasingly acknowledged. Xanthos, Sandkühler, and other researchers introduced the term "neurogenic neuroinflammation [20]." In the context of migraines, neurogenic neuroinflammation refers to the inflammatory response in both the central and peripheral components of the trigeminal nervous system to neuronal activity. Edvisson L and his colleagues suggest that the inflammatory process triggered by the continuous release of neurotransmitters may contribute to the chronicity of migraines [21]. Nucleus factor erythroid 2-related factor 2 (Nrf2) is a transcription factor that is widely expressed in the central nervous system [22]. The study demonstrates that activating the Nrf2 signaling pathway can reduce oxidative stress and neuroinflammation [23, 24]. Other related research has shown that activation of the Nrf2/ARE pathway attenuates nociceptive sensitization in NTG-induced migraine mice [25, 26], suggesting that the Nrf2/ARE pathway may be a new therapeutic target for migraine.

DL-3-n-butylphthalide (NBP), a small-molecule compound independently developed from celery seed in China, was approved by the Chinese State Food and Drug Administration (SFDA) in 2005 for the treatment of ischemic stroke. It is relatively inexpensive with a very low incidence of adverse effects [27]. Numerous studies have documented that NBP exhibits the potential to mitigate oxidative stress and neuroinflammation through the activation of the Nrf2 pathway, with observed therapeutic benefits in animal models of Alzheimer's disease and depression [28, 29]. Nevertheless, it remains unexplored whether NBP can extend its therapeutic efficacy to the treatment of migraine.

In this study, we established a chronic migraine mouse model and administered NBP pre-treatment to simulate prophylactic treatment. Through this methodology, we hoped to evaluate the therapeutic effects of NBP through the behavioural performance of the mice, the expression levels of migraine-related markers, as well as oxidative stress and inflammatory factors. In addition, we also analyzed the Nrf2 pathway to further understand the mechanism of NBP treatment of migraine.

## Results

### Recurrent administration of NTG induces migraine-like behaviors and upregulates the expression of CGRP and c-Fos in SP5C

**Fig. 1:**
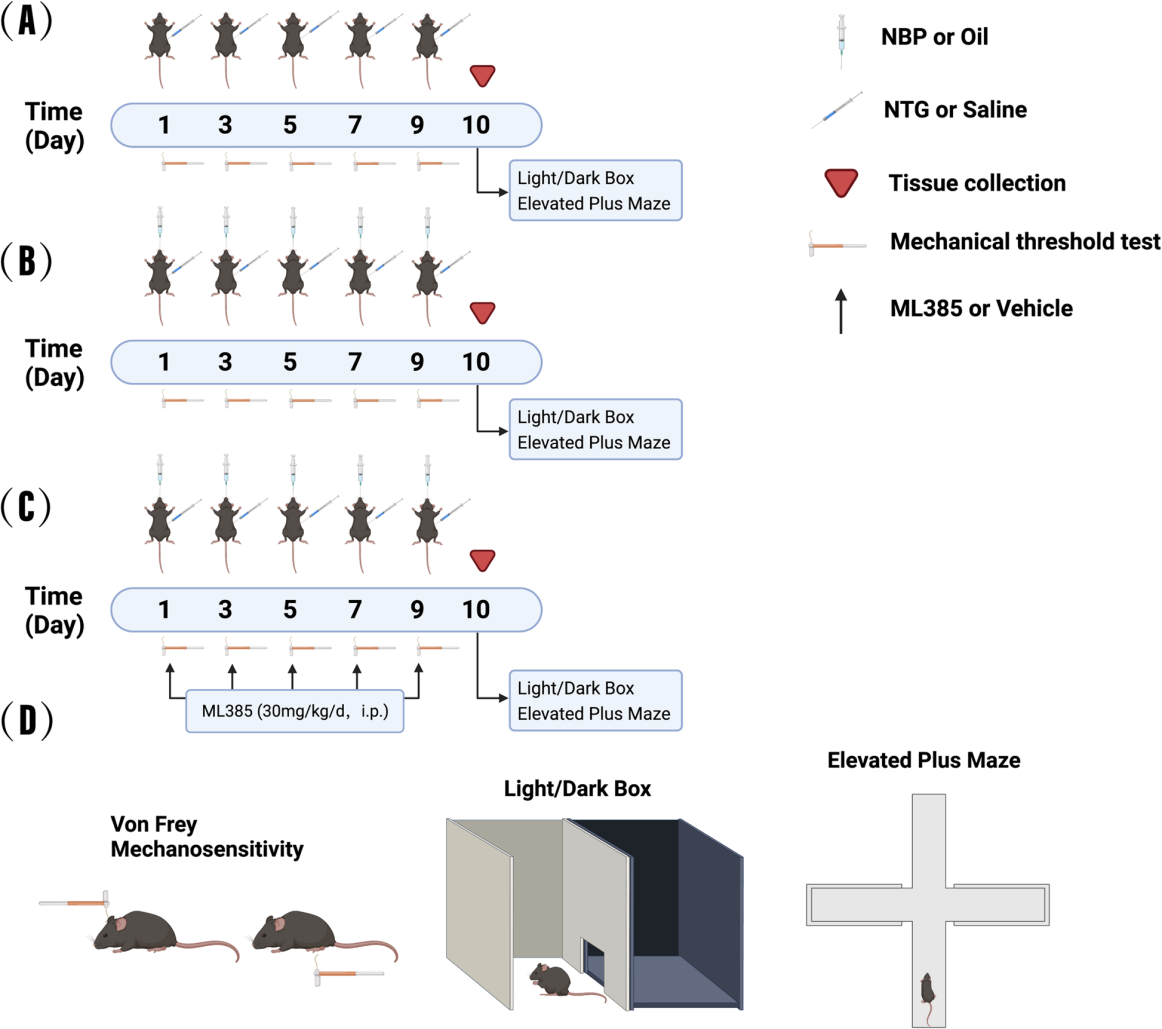
Timing of drug administration, behavioral testing and sampling, and behavioral paradigms. **A** The Control and NTG groups. **B** The Control + Oil, NTG + Oil and NTG + NBP groups. **C** The Control + Oil + ML385, NTG + Oil + ML385, NTG + NBP + ML385 and NTG + NBP + Vehicle groups. Tissue collection was performed within 24 h after the last NTG injection, photophobia, and elevated-plus maze experiments were performed within 24 h after the last NTG injection, and mechanical thresholds were performed 30 min before each NTG injection. **D** Schematic representation of mechanical threshold, photophobia, and anxiety behaviour tests

**Fig. 2:**
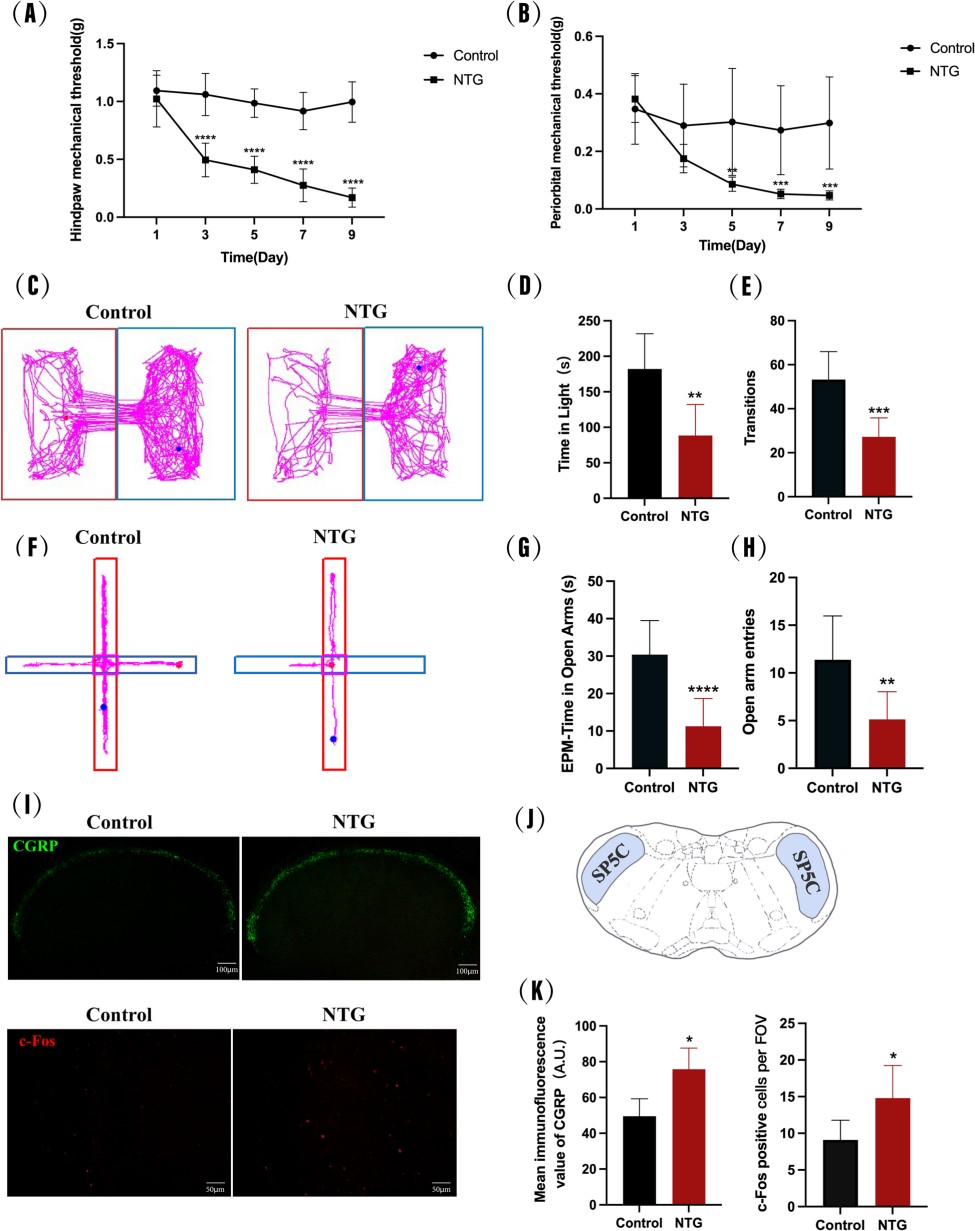
Recurrent NTG injection induced hyperalgesia and upregulated the expression of CGRP, c-Fos. **A**, **B** Periorbital and paw mechanical threshold during the injection of NTG. **C**, **D**, **E** Diagram showing the mouse's trajectory in the light and dark boxes, the time spent in the light box, and the number of times it entered the lightbox. **F**, **G**, **H** Mouse trajectory graph in EPM, time spent in open arm, and number of entries in open arm. **I**, **J** Immunofluorescence staining images of CGRP and c-Fos in the SP5C. (K) Location of SP5C. (*n* = 6–8 in each group; scale bar = 50/100 μm; *p < 0.05, **p < 0.01, ***p < 0.001 and ****p < 0.0001 compared with the Control group)

Based on previous reports [30], a chronic migraine model was constructed by repeatedly administering intraperitoneal injections of NTG for a total of five doses on alternate days, with the first day of administration being designated as day 1 (Fig. 1A). To investigate migraine-like behaviors in mice, cutaneous allodynia, photophobia and anxiety-like behaviors were evaluated by mechanical threshold test, light-aversive test and elevated plus maze (EPM) test, respectively. Mechanical threshold test was performed 30 min before each NTG injection. Light-aversive test and EPM test were conducted 24 h after the final NTG dose. We observed that the NTG group exhibited significant and time-dependent decreases in both hindpaw and periorbital mechanical thresholds compared to the Control group, starting from day 3 and reaching the lowest levels on day 9 (Fig. 2A-B). In the light-aversion test, we found that NTG mice, compared with Control mice, spent significantly less time in the light chamber and made fewer transitions between the two chambers during the 10-min test period (Fig. 2C-E). In the EPM test, we found that repeated NTG injection reduced the time spent in open arms and the number entering to open arm of mice (Fig. 2F -H). These results indicate that NTG-treated mice exhibited cutaneous allodynia, photophobia and increased anxiety-like behavior after repeated NTG treatment.

Upregulated expression of CGRP and c-Fos in the SP5C were observed in several studies and considered to be related to the central sensitization in mice model of migraine. Our immunofluorescence staining showed that repeated administration of NTG significantly increased the expression of CGRP and c-Fos in the SP5C, and both were statistically significantly different from the Control group (Fig. 2I-K). These data indicate that the CM model we have constructed is reliable and can be used for further studies.

### NBP alleviates recurrent NTG-induced migraine-like behaviors

**Fig. 3:**
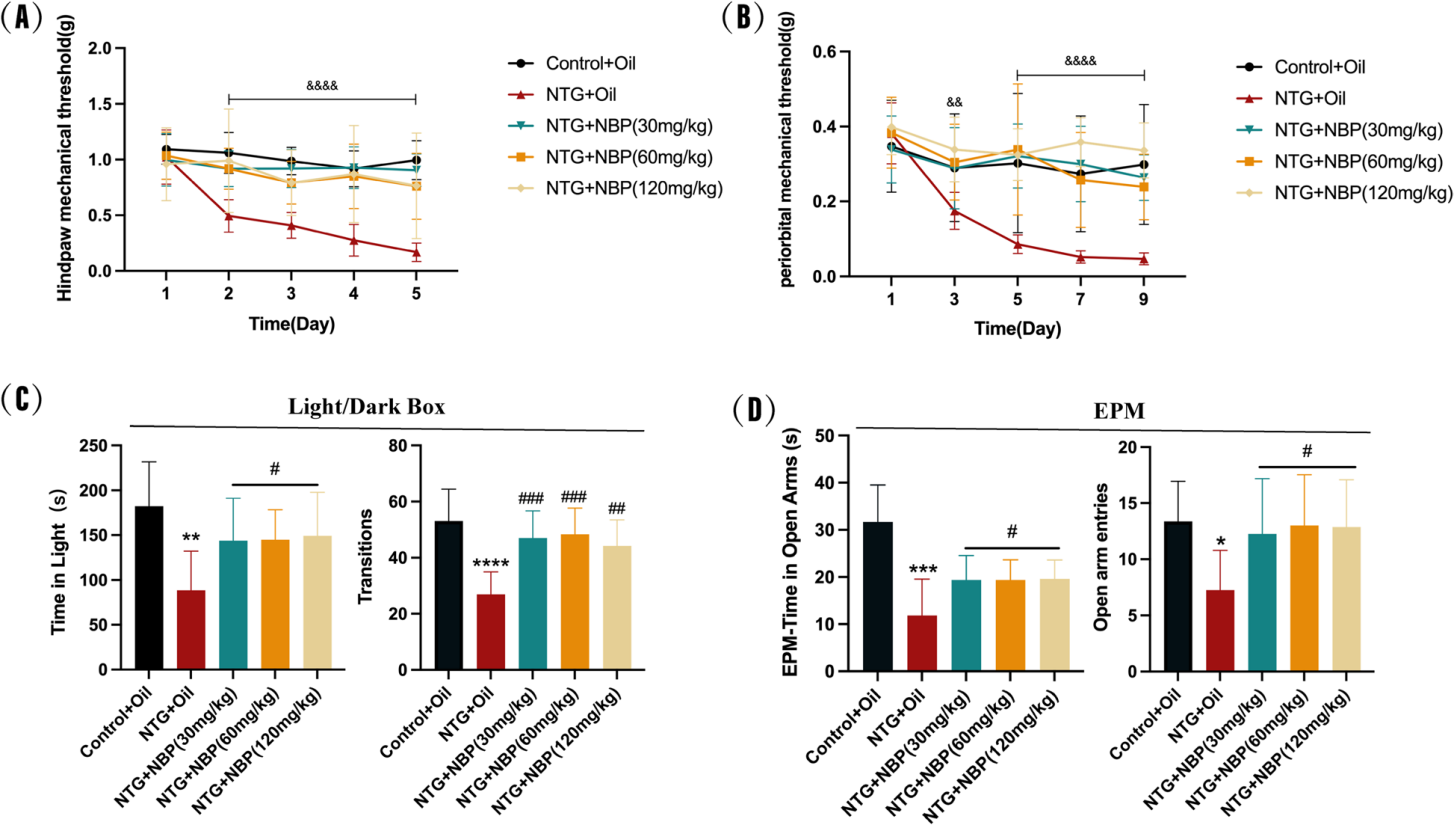
Effects of NBP on mechanical thresholds, photophobia, and anxiety. **A**, **B** Compared with the NTG + Oil group, NBP at 30 mg/kg, 60 mg/kg, and 120 mg/kg significantly protected the hindpaw and periorbital mechanical thresholds in mice. **C** Compared with the NTG + Oil group, 30 mg/kg, 60 mg/kg, and 120 mg/kg NBP significantly increased the time spent in the light box and the number of times the mice entered the lightbox. **D** Compared with the NTG + Oil group, 30 mg/kg, 60 mg/kg and 120 mg/kg of NBP significantly increased the time spent in the open arm and the number of entries into the open arm. (*n* = 8–10 in each group; *p* < 0.01,*p* < 0.0001 the NTG + NBP (30mg/kg) group compared with the NTG + Oil group; *p < 0.05, **p < 0.01, ***p < 0.001 and ****p < 0.0001 compared with the Control + Oil group; #*p* < 0.05, ##*p* < 0.01, ###*p* < 0.001 compared with the NTG + Oil group)

To investigate the effect of NBP on NTG-induced migraine-like behaviors, we intragastrically administered three different doses of NBP (30 mg/kg, 60 mg/kg and 120 mg/kg) 15 min before each NTG injection and assessed the cutaneous allodynia, photophobia and anxiety-like behaviors of mice as described above (Fig. 1B). Consistent with our results in Fig. 2, NTG treated mice exhibited allodynia, photophobia and anxiety-like behaviors compared to Control mice (Fig. 3). Excitingly, pre-treatment of all three dosage of NBP robustly prevented the decrease of hindpaw and periorbital mechanical thresholds (Fig. 3A-B), time spent in light and the number of transitions between the two chambers (Fig. 3C), time spent in open arms and the number of open arm entries in NTG mice (Fig. 3D). Additionally, our data did not reveal any substantial disparities in migraine-like behavioral improvement among three dosages of NTG + NBP groups. These data suggest a therapeutic effect of pre-treatment of NBP on migraine-like behaviors in NTG mice. To avoid potential toxicity from high doses of NBP and since all three doses had similar effects, we chose the 30 mg/kg dose for further study.

### NBP reduced recurrent NTG-induced upregulated expression of CGRP and c-Fos in the SP5C

**Fig. 4:**
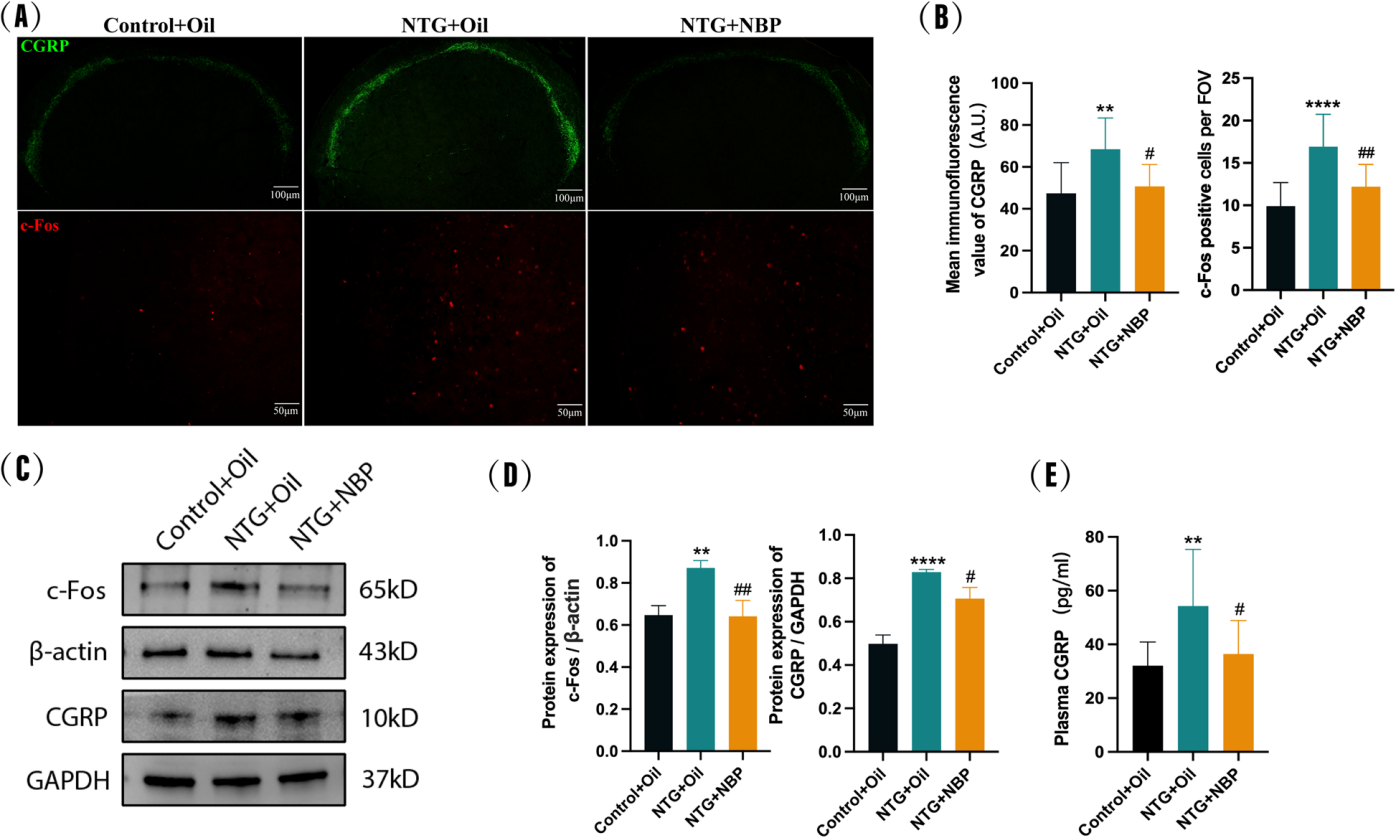
Effect of NBP on CGRP and c-Fos expression in SP5C. **A**, **B** CGRP and c-fos immunofluorescence staining. CGRP immunofluorescence intensity and the number of c-fos-positive cells were significantly increased in the NTG + Oil group compared with the Control + Oil group, and CGRP immunofluorescence intensity and the number of c-fos-positive cells were significantly decreased in the NTG + NBP group compared with the NTG + Oil group. **C**, **D** Protein expression of CGRP and c-Fos. CGRP and c-Fos protein levels were significantly higher in the NTG + Oil group compared to the Control + Oil group. CGRP and c-Fos protein levels were significantly lower in the NTG + NBP group compared to the NTG + Oil group. **E** Expression of CGRP in plasma. Plasma CGRP expression was significantly higher in the NTG + Oil group compared to the Control + Oil group and significantly lower in the NTG + NBP group compared to the NTG + Oil group. (*n* = 6–8 in each group; scale bar = 50/100 μm; **p < 0.01 and ****p < 0.0001 compared with the Control + Oil group; #*p* < 0.05 and ##*p* < 0.01 compared with the NTG + Oil group)

To further explore whether the therapeutic effect of NBP is related to the expression of CGRP and c-Fos in the SP5C, we performed the CGRP and c-Fos immunofluorescence experiment after treating NTG mice with NBP. As shown in Fig. 3A and B, the expression of CGRP and c-Fos in the SP5C of NTG mice was upregulated compared to Control mice, consistent with our previous results in Fig. 2. However, pre-treatment of NBP significantly reduced the increased expression of CGRP and c-Fos in the SP5C of NTG mice (Fig. 3A and B). The western blot experiment also confirmed that NBP decreased NTG-induced upregulation of CGRP and c-Fos in the SP5C (Fig. 4 C and D), reinforcing the important role of NBP in regulating the expression of CGRP and c-Fos in the SP5C of NTG mice. Moreover, the plasma CGRP levels of the mice showed a similar trend as in the SP5C (Fig. 4E).

### NBP alleviates recurrent NTG-induced oxidative stress in the SP5C

**Fig. 5:**
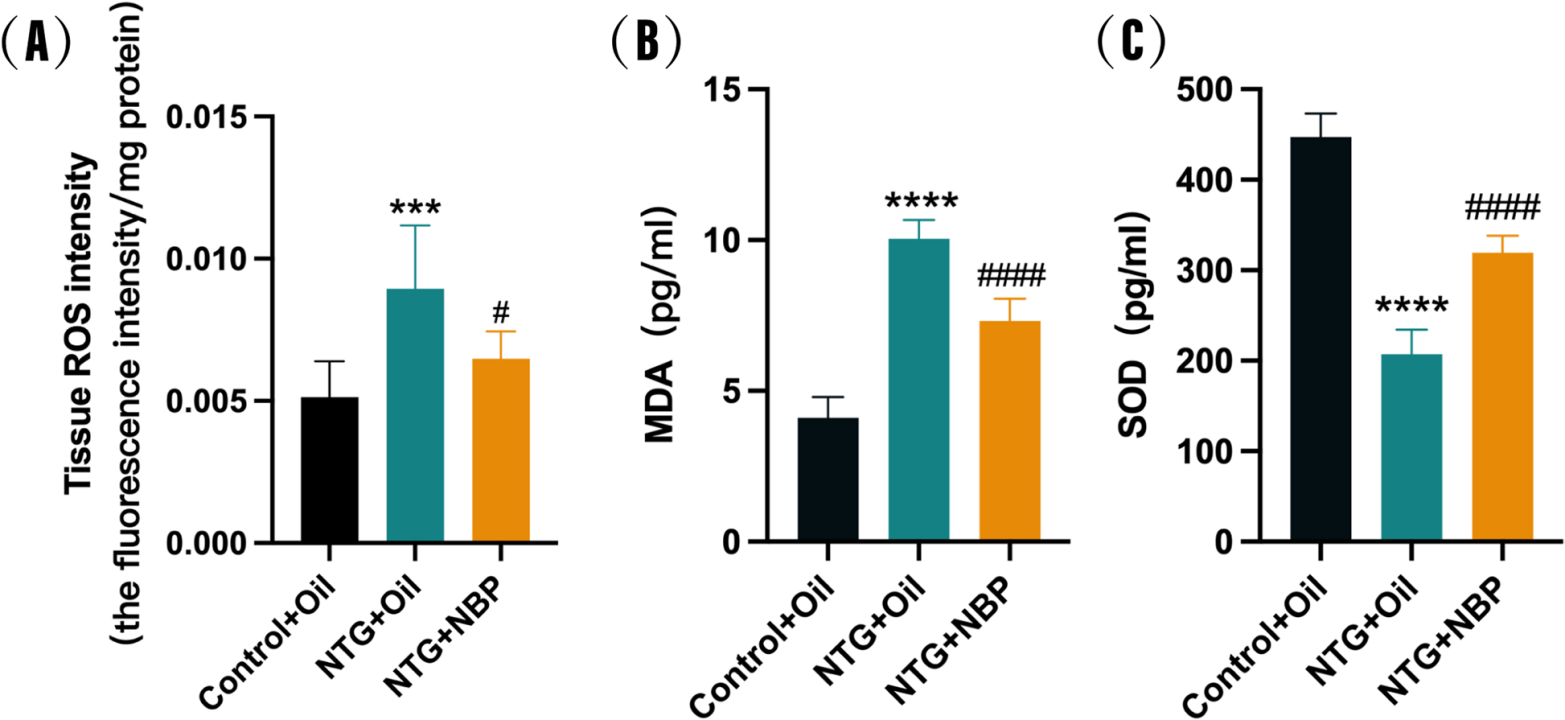
Effect of NBP on the level of oxidative stress in the SP5C. **A** ROS levels were significantly increased in the SP5C of the NTG + Oil group compared to the Control + Oil group and significantly decreased in the NTG + NBP group compared to the NTG + Oil group. **B** MDA levels were significantly higher in the SP5C of the NTG + Oil group compared to the Control + Oil group and significantly lower in the NTG + NBP group compared to the NTG + Oil group. **C** SOD levels were significantly decreased in the SP5C of the NTG + Oil group compared to the Control + Oil group and significantly increased in the NTG + NBP group compared to the NTG + Oil group. (*n* = 6–8 in each group; ***p < 0.001 and ****p < 0.0001 compared with the Control + Oil group; #*p* < 0.05 and ####*p* < 0.0001 compared with the NTG + Oil group)

Oxidative stress causes excessive production of reactive oxygen species (ROS), which is believed to be intricately involved in the pathophysiological mechanisms of migraine. The ROS can damage lipids and generate cytotoxic malondialdehyde (MDA). SOD stands as an essential antioxidant enzyme responsible for scavenging oxygen free radicals. To determine whether the therapeutic effect of NBP is derived from its ability to alleviate oxidative stress, we examined the expression levels of ROS, MDA and SOD in the SP5C. We observed that the NTG + Oil group exhibited elevated levels of ROS and MDA (Fig. 5A and B), which were significantly different from the Control + Oil group. However, the levels of ROS and MDA in the NTG mice treated with NBP were significantly reduced compared with the NTG + Oil group. In addition, as shown in Fig. 5C, SOD was significantly lower in the NTG + Oil group compared with the Control + Oil group, and the NBP intervention could attenuate this reduction. Taken together, these results demonstrate that the antioxidant effect of NBP may be related to its therapeutic effect in NTG mice.

### NBP inhibits recurrent NTG-induced inflammation in the SP5C

**Fig. 6:**
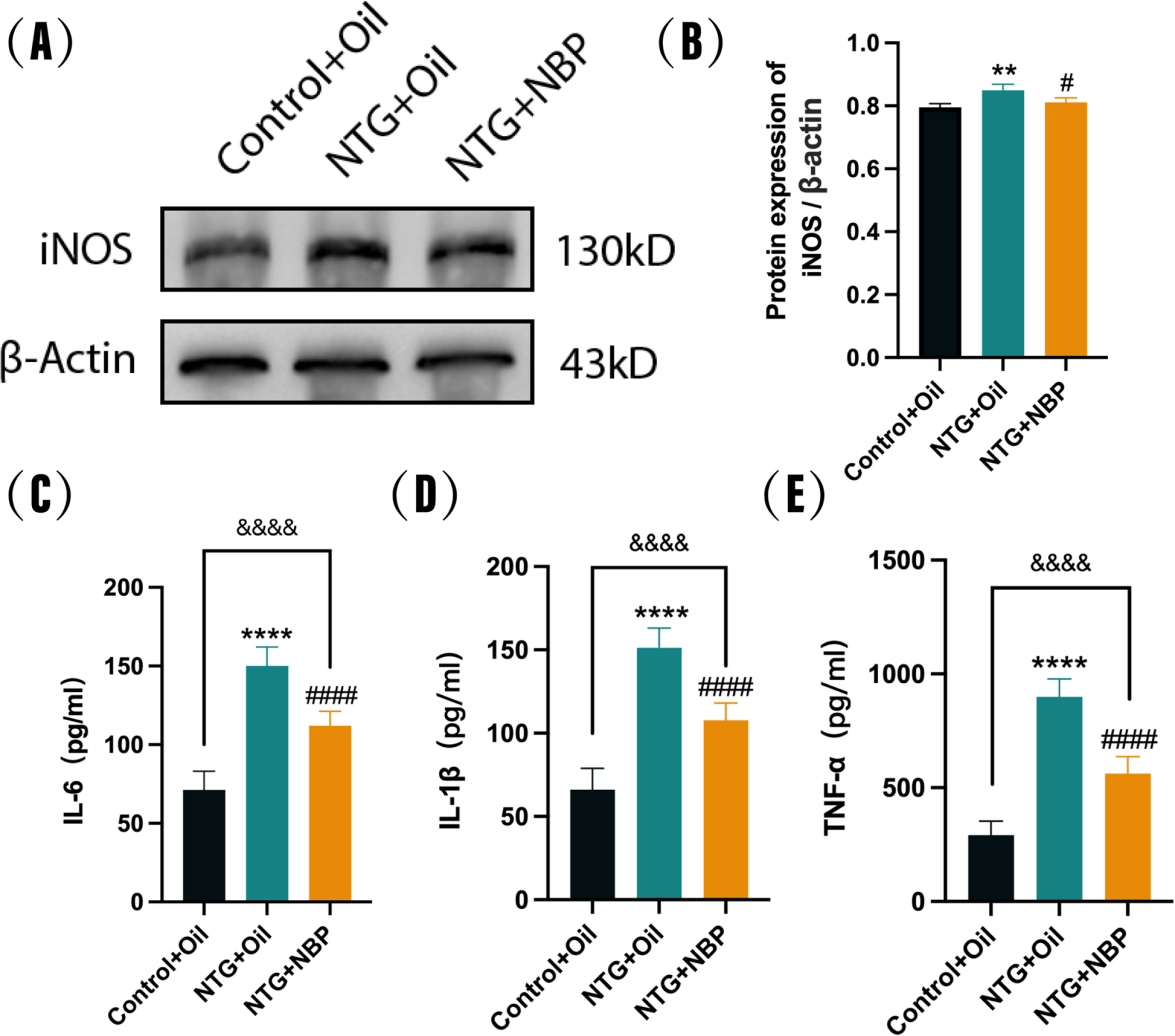
Effect of NBP on neuroinflammation in the SP5C. **A**, **B** Expression of iNOS proteins. iNOS protein expression was increased in the SP5C in the NTG + Oil group compared with the Control + Oil group and in the NTG + NBP group compared with the NTG + Oil group. **C**, **D**, **E** IL-1β, IL-6 and TNF-α expression was increased in the SP5C in the NTG + Oil group compared with the Control + Oil group, and IL-1β, IL-6 and TNF-α protein expression was reduced in the NTG + NBP group compared with the NTG + Oil group. (*n* = 6–8 in each group; **p < 0.01 and ****p < 0.0001 compared with the Control + Oil group; #*p* < 0.05 and ####*p* < 0.0001 compared with the NTG + Oil group)

Neurogenic inflammation plays a key role in the pathophysiology and chronicity of migraine. We further explored the inhibitory effect of NBP on NTG-induced inflammation by detecting the inflammation-related indicators iNOS, IL-1β, IL-6, and TNF-α in SP5C. WB results showed that repeated administration of NTG significantly increased iNOS levels in the SP5C compared to the Control + Oil group, and pre-treatment with NBP reversed this change (Fig. 6A-B). Meanwhile, as depicted in Fig. 6C-E, levels of inflammatory factors such as IL-1β, IL-6, and TNF-α were elevated in NTG mice compared to those in the Control + Oil group. However, treatment with NBP significantly attenuated the upregulation induced by recurrent NTG administration, with the NTG + NBP group showing statistically significant differences compared to the Control + Oil group. These findings indicate that NBP exerts a partial inhibitory effect on SP5C neuroinflammation in NTG mice, potentially contributing to its therapeutic impact on migraine-like behavior.

### NBP activates Nrf2 inhibited by recurrent NTG administration and increases its downstream protein expression

**Fig. 7:**
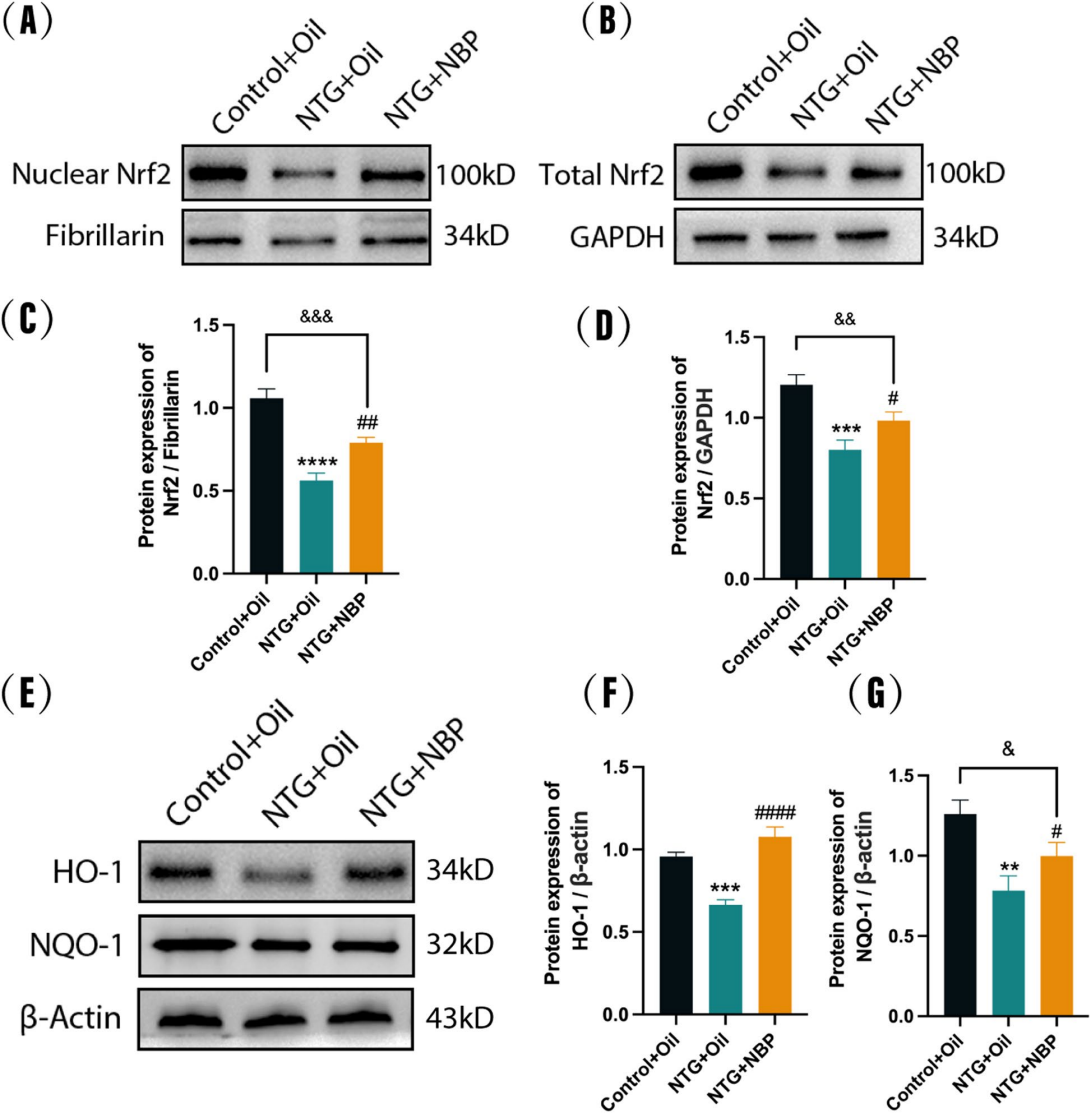
Effect of NBP on Nrf2 protein expression levels in nuclear and total cells of SP5C and HO-1, NQO-1 protein expression levels. **A**, **B**, **C**, **D** Nuclear and total cellular Nrf2 protein expression was decreased in the NTG + Oil group compared to the Control + Oil group, and increased in the NTG + NBP group compared to the NTG + Oil group. **E**, **F**, **G** HO-1 and NQO-1 protein expression was decreased in the NTG + Oil group compared to the Control + Oil group and increased in the NTG + NBP group compared to the NTG + Oil group. (*n* = 3–5 in each group; **p <0.01, ***p < 0.001 and ****p < 0.0001 compared with the Control + Oil group; #*p* < 0.05, ##*p* < 0.01 and ####*p* < 0.0001 compared with the NTG + Oil group)

There is considerable evidence indicate that the Nrf2-ARE pathway plays a protective role in neurological disorders by reducing oxidative stress and neuroinflammation [24]. To explore the role of Nrf2 pathway underlying the mechanism of NBP exerting its antioxidant and anti-neuroinflammation effect in NTG mice, we analyzed the expression levels of Nrf2 in the nucleus and total cells of the SP5C. Results showed that repeated administration of NTG significantly reduced the expression of cytosolic Nrf2 and total Nrf2 compared to the Control + Oil group, while pre-treatment with NBP significantly reversed the downregulation of Nrf2 (Fig. 7A-D). In line with the downregulated Nrf2, the levels of downstream proteins including HO-1 and NQO-1 were apparently lower in the NTG + Oil group than those in the Control + Oil group. Meanwhile, A significant upregulation of HO-1 and NQO-1 was observed in the NTG + NBP group compared with the NTG + Oil group (Fig. 7E-G). In addition, we also found that in these data, the NTG + NBP group still has statistical differences compared to the Control + Oil group. Taken together, our results indicate that NBP may exert antioxidant and anti-neuroinflammation effect in NTG mice by partial activating the Nrf2 pathway.

### Inhibition of Nrf2 blocks the improvement of NBP on migraine-like behavior in NTG mice

**Fig. 8:**
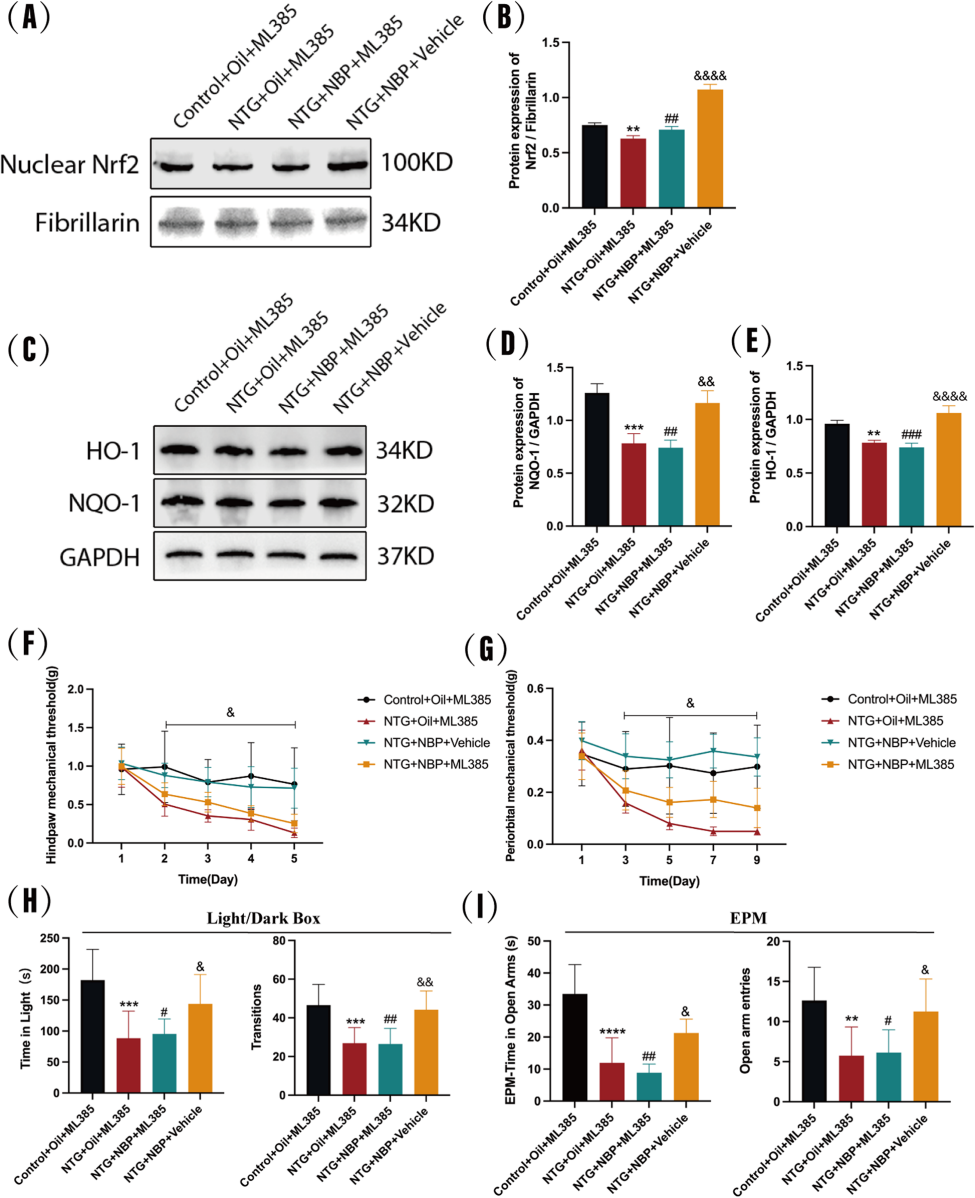
Effect of ML385 on the expression of Nrf2 and its downstream proteins in TNC. **A, B** Nrf2 protein expression in the nuclear of the TNC was reduced in the NTG+Oil+ML385 group compared to the Control+Oil+ML385 group, and there was no significant change in Nrf2 protein expression in the NTG+NBP+ML385 group compared to the NTG+Oil+ML385 group, whereas Nrf2 protein expression was significantly increased in the NTG+NBP+Vehicle group. **C, D, E** The expression of HO-1 and NQO-1 proteins in the TNC was reduced in the NTG+Oil+ML385 group compared to the Control+Oil+ML385 group, and there was no significant change in the expression of HO-1 and NQO-1 proteins in the NTG+NBP+ML385 group compared to the NTG+Oil+ML385 group, whereas the expression in the NTG+NBP+Vehicle group was significantly increased. (n = 3–5 in each group; **p < 0.01 and ***p < 0.001 compared with the Control+Oil+ML385 group; ##p < 0.01 and ###p < 0.001 compared with the NTG+NBP+Vehicle group; &&p < 0.01 and &&&&p < 0.0001 compared with the NTG+Oil+ML385 group). **F, G** Inhibition of Nrf2 by ML385 deprives NBP of protection against mechanical thresholds. The NTG+NBP+ML385 group did not improve the decrease in mechanical thresholds and there was no difference in the NTG+NBP+ML385 group compared to the NTG+Oil+ML385 group. However, the NTG+NBP+vehicle group significantly protected against mechanical nociceptive sensitization compared to the NTG+Oil+ML385 group. (n = 8–10 in each group; &p < 0.05 NTG+NBP+ML385 compared with the NTG+NBP+Vehicle group). As shown in **H** and **I**, there was no difference between the NTG+Oil+ML385 group and the NTG+NBP+ML385 group. Photophobic and anxious behaviours were not improved in the NTG+NBP+ML385 group compared with the NTG+NBP+Vehicle group. (n = 8–10 in each group; **p < 0.01, ***p < 0.001 and ****p < 0.0001 compared with the Control+Oil+ML385 group; ##p < 0.01 and ###p < 0.001 compared with the NTG+NBP+Vehicle group; &p < 0.05 and &&p < 0.01 the NTG+NBP+Vehicle group compared with the NTG+Oil+ML385 group)

ML385 is a specific inhibitor of Nrf2, and previous studies have shown that intraperitoneal administration of ML385 at 30 mg/kg can significantly inhibit Nrf2 activation in mice [31, 32]. In order to further clarify the relationship between activation of Nrf2 pathway and the therapeutic effect of NBP on migraine-like behavior in NTG mice, we employed ML385 to inhibit the Nrf2 pathway and assessed its effect on the migraine-like behaviors in NBP treated mice (Fig. 1C). The experimental findings indicated a significant reduction in nuclear Nrf2, HO-1 and NQO-1 expression in the SP5C following ML385 administration in comparison to the NBP-treated group (Fig. 8A-E). This observation implies that ML385 effectively inhibited the activation of the Nrf2 pathway in NTG + NBP mice.

Notably, behavioral experiments demonstrated that ML385 did not exert a substantial impact on the behavior of mice within both the Control + Oil + ML385 and NTG + Oil + ML385 groups. In contrast, administration of ML385 significantly reversed the improvement of NBP on the reduction of hindpaw and periorbital mechanical thresholds in NTG mice compared to the NBP-treated group (Fig. 8F-G). Meanwhile, ML385 reversed the increase of time spent in light, the number of transitions between the two chambers (Fig. 8H), time spent in open arms and the number of open arm entries in NTG + NBP mice (Fig. 8I). Overall, we found that blocking the Nrf2 pathway significantly attenuated the therapeutic effect of NBP, which indicate that the alleviation of NBP on migraine-like behavior in NTG mice is dependent on the Nrf2 pathway.

## Discussion

In the present study, we first developed an NTG-induced chronic migraine mouse model and confirmed the model’s reliability by measuring migraine-like behaviors and the abnormal expression of c-Fos and CGRP in the SP5C. Subsequently, we discovered that NBP pretreatment significantly ameliorated NTG-induced migraine-like behaviors and mitigated the increased expression of c-Fos and CGRP triggered by repeated NTG administration in mice. Meanwhile, NBP alleviated the enhanced oxidative stress and inflammation in the SP5C in NTG mice. Our experimental results further verified that NBP activates Nrf2 and its downstream targets. The administration of ML385 rendered NBP ineffective in alleviating migraine-like behaviors in NTG mice, indicating that the therapeutic efficacy of NBP hinges on the activation of the Nrf2/ARE pathway. Our research affirms that NBP activation of the Nrf2 pathway alleviates central sensitization in chronic migraine mice by mitigating oxidative stress and neuroinflammation.

NTG-treated mice are now a well-established and widely used experimental model of migraine, which can mimic many clinical features of migraine patients [33–35]. Repeated injections of NTG resulted in a gradual decrease in mechanical nociceptive thresholds, an effect that was observed for up to one week following the cessation of NTG [33]. This hypersensitivity is indicative of cutaneous allodynia, which is present not only in the head but also in other areas of the body [36, 37]. In our present study, both periorbital and hindpaw cutaneous allodynia were observed, which suggest a severe state of central sensitization in NTG-treatment mice. Central sensitization is considered an important mechanism for the chronicity of migraine. Moreover, about 80% of migraineurs have concomitant symptoms of photophobia, and anxiety and depression [38]. Harris et al. have demonstrated photophobia and anxiety-like behaviour in NTG-induced animal models [39]. Here, we similarly assessed photophobia and anxiety-like behaviour to confirm the reliability of the model.

Photophobia is one of the most common symptoms of migraine, and the underlying mechanism is uncertain. The coexistence of photophobia and headache is associated with the interactions between visual and pain pathway at retina, midbrain, thalamus, hypothalamus and visual cortex. The communication between these pathways may depend on CGRP and pituitary cyclase-activating polypeptide transmission [40]. However, in our study, NBP improved the photophobic performance of CM mice, which may be achieved by lowering CGRP levels. Migraine is associated with a variety of psychiatric co-morbidities, including anxiety-depression, and the mechanisms involved are unclear [41]. Ruozhi Dang et al. reported that Edaravone attenuated oxidative stress through the Nrf2 pathway and alleviated anxiety-depressive behaviour in mice [42]. This may be consistent with the pathway by which NBP alleviated anxiety-depressive behaviour in CM mice in the present study.

The role of CGRP in migraine is well-established. It enhances nociceptive transmission and contributes to central sensitization by fostering and maintaining a hyper-responsive state [43]. The nuclear protein c-Fos is rapidly expressed in neurons in response to various types of noxious stimuli [44]. A study conducted by Shouyi Wu et al. evaluated NTG-induced migraine in mice by quantifying c-Fos and CGRP levels in SP5C and assessing central sensitization [45]. In our current study, we adopted a similar methodology to evaluate migraine, with an added focus on the expression levels of CGRP in peripheral blood. Our findings revealed that the changes in peripheral CGRP mirrored those observed in the central nervous system, corroborating the elevated CGRP levels found in the blood of migraine patients [9].

Within the known pathogenesis of migraine, oxidative stress and inflammation have been identified as universal triggers [11, 46, 47]. Riboflavin, known for its ability to reduce oxidative stress, has been found to prevent migraines [47]. The link between migraines and inflammation is still a hypothesis and has not been definitively confirmed. However, a growing body of research indicates a potential connection between inflammation and migraines. Numerous experiments, primarily conducted on rodent models, suggest that the nociceptive trigeminocervical complex, responsible for headaches, can be triggered by aseptic meningeal inflammatory processes [48]. Additionally, a study by Wei He et al. suggests that the activation of NLRP3 in microglia plays a role in the inflammatory responses associated with central sensitization of migraines in the NTG-induced CM mouse model [45]. Research by Rosaria Greco et al. demonstrates increased expression of pro-inflammatory cytokines in the TNC region of rats in the NTG-induced migraine model [49]. Furthermore, a PET-MRI study utilizing a highly sensitive inflammation tracer on 11 migraine patients has confirmed the connection between meningeal inflammation and migraines [50]. These collective findings suggest that although the precise relationship between migraines and inflammation remains unclear, inflammation may indeed contribute to the development of migraines. Consequently, strategies that target oxidative stress and inflammation present a viable approach for migraine management. Nrf2 is increasingly recognized as a key regulator of cellular defenses against oxidative stress. The activation of the Nrf2 signaling pathway serves to protect cells from oxidative stress and inflammation [24].

In China, NBP has been approved for the treatment of cerebral ischemia. However, numerous researchers have discovered that NBP also holds therapeutic potential in various neurological disorders. Mengqi Yang et al. showed that NBP may be able to treat major depressive disorder (MDD) [28]. Chun-Yan Wang et al. showed that NBP treatment could suppress TXNIP-NLRP3 interaction and inhibit NLRP3 inflammasome activation via upregulation of Nrf2 [51]. These findings suggest that NBP may have a role in activating Nrf2 expression and ameliorating the level of oxidative stress and inflammation, and in the present study, we found that NBP may ameliorate migraine through the same mechanism of action. Similarly, Wei Di et al. found that activation of the Nrf2 pathway ameliorates NTG-induced migraine in mice, which is consistent with our findings. They also found in a single administration of NTG-induced migraine mouse model, the expression of Nrf2 in the nucleus of cells in the SP5C was significantly increased compared to the Control + group [25], which may be due to the fact that NTG injections may enhance the antioxidant capacity of cells to cope with nitric oxide-induced oxidative stress by inducing the expression of Nrf2, causing a compensatory mechanism. However, our results showed that repeated NTG injections led to a decrease in nuclear Nrf2 expression, which may be due to the fact that multiple NTG injections mimic the chronicity of migraine, in which the body's antioxidant capacity is insufficient to combat peroxidation, resulting in a state of loss of compensation. In addition, some studies of other chronic neurological disorders, such as Amyotrophic Lateral Sclerosis (ALS) and MDD, have also shown a decrease in Nrf2 expression in the animal model group [28, 52], which is consistent with our findings.

Furthermore, several studies have shown that the Nrf2/ARE pathway not only regulates oxidative stress but also attenuates inflammation [24, 53, 54], as the HO-1 axis regulated by Nrf2 is a potent anti-inflammatory target [53]. During our experiments, we found that NBP attenuated the high expression of pro-inflammatory factors and improved some indicators of oxidative stress. These results suggest that NBP may alleviate migraine in mice by reducing oxidative stress and neuroinflammation, but the specific anti-inflammatory mechanism of Nrf2 needs further investigation. ML385, a cancer-fighting molecule commonly used in tumor research, specifically inhibits the binding of Nrf2 to DNA, thereby suppressing the expression of Nrf2 [55], and previous studies have shown that intraperitoneal administration of ML385 at 30 mg/kg can significantly inhibit Nrf2 activation in mice [31, 32]. After administration of ML385 to block Nrf2 expression, we found that NBP failed to improve migraine-like behavioural in mice, which may indicate that NBP treatment is dependent on the Nrf2/ARE pathway. Nonetheless, it is important to address one of the limitations of our study, which is that we did not further substantiate this idea by deeper molecular studies. This is something we intend to focus on in our future and complementary studies. Meanwhile, the application of ML385 had no effect on the behaviour of normal mice, which may be due to the fact that the level of oxidative stress in normal mice is at a plateau, and inhibition of Nrf2 does not affect the normal level of oxidative stress.

In summary, our study highlights the significant preventive therapeutic potential of NBP in a mouse model of chronic migraine. This suggests that NBP could serve as an effective agent for preventing migraine attacks, a prospect that is particularly promising given the limitations of existing prophylactic medications for migraine. Moreover, the anti-inflammatory and antioxidant properties of NBP, as demonstrated in our study, suggest that NBP could also be effective in the acute treatment of migraine and other pain disorders. This possibility is intriguing and warrants further investigation in our future research. In conclusion, our study not only provides a effective strategy for managing migraines, but also broadens the potential applications of NBP in other medical conditions.

## Conclusion

In summary, our study demonstrated that NBP effectively attenuated NTG-induced migraine in mice. It exerted its therapeutic effects by mitigating nociceptive hypersensitivity and central sensitization through the activation of the Nrf2 signaling pathway, thereby reducing oxidative stress and neuroinflammation. Consequently, NBP holds promising potential as a prophylactic treatment for migraine.

## Methods

### Animals

Male mice were used throughout the study to eliminate possible hormonal cycle effects. SPF-grade wild-type (C57BL/6 J) mice (weighing 18–20 g, aged 6–8 weeks) were purchased from SiPeiFu Biotechnology Co, Ltd (Beijing, China). We maintained all the animals under standard conditions of 22 ± 2°C, 50 ± 10% relative humidity, alternating light, and dark cycles for 12 h, and provided them with adequate food and water. Every effort was made to minimize the number of animals used in the experiment and to minimize animal suffering. Prior to the experiment, all animals were allowed to acclimatize for one week and were then randomly assigned to different experimental groups according to a sequence of random numbers generated by Excel software. During the treatment period, the animals were weighed daily and no adverse effects were observed (Fig. S1).

All experimental procedures were approved by the Institutional Animal Care and Use Committee of the Chinese People's Liberation Army (PLA) General Hospital, and animal experiments were conducted according to the Animal Research: Reporting of In Vivo Experiments (ARRIVE) guidelines. Details of the procedures (drug administration, behavioural testing, sample collection) are show in Fig. 1.

### Establishment of the chronic migraine model

Nitroglycerine (NTG) is the most common and recognized experimental migraine trigger in humans and rodents. NTG induces migraine-like attacks with nociceptive hypersensitivity and striatal site aversion [35]. We used NTG to investigate potential mechanisms associated with migraine. NTG (5 mg/ml in ethanol, Beijing Yimin Pharmaceutical Co., Ltd., Beijing, China) was freshly diluted in 0.9% (wt /vol) saline to a final concentration of 0.5 mg/ml for a dose of 10 mg/kg. In brief, the chronic migraine model was simulated by an intraperitoneal injection every other day (five injections in total), each at a dose of 10 mg/kg.ce.

### Drug administration

According to the experimental requirements, mice were randomly divided into the following groups: (1) Control group, (2) NTG group, (3) Control + Oil group, (4) NTG + Oil group, (5) NTG + NBP (30mg/kg) group, (6) NTG + NBP (60mg/kg) group, (7) NTG + NBP (120mg/kg) group, (8) Control + Oil + ML385 group, (9) NTG + Oil + ML385 group, (11) NTG + NBP + Vehicle group, (12) NTG + NBP + ML385 group, NBP (dis-solved in soybean oil, provided by Shijiazhuang PharmaGroup NBP Pharmaceutical Co. Ltd., Shijiazhuang, Hebei Province, China). For simulated preventive treatment, NBP or an equivalent amount of solvent was administered by gavage 5 min before each NTG injection. The dose of NBP was derived from previous literature, these studies show that the drug is neuroprotective [28, 56, 57].

ML385 (Medchem Express, Monmouth Junction, New Jersey, USA) is a specific inhibitor of Nrf2, often used to reduce Nrf2 expression in mice. Mice were injected intraperitoneally with 30 mg/kg ML385 (dis-solved in saline containing 50% PEG300) 30 min before each NTG injection or equivalent amount of solvent. The dose of ML385 was determined from the literature [31].

### Sensory sensitivity testing

Central sensitization stands as a pivotal mechanism contributing to the chronic nature of migraine. This phenomenon is believed to transpire sequentially within second and third-level neurons, culminating in the development of cutaneous abnormal pain with lateral and extracranial symptoms, which exhibit a similar spatial distribution. In light of this, we conducted an assessment of periorbital and hindpaw nociceptive hypersensitivity within the NTG mouse model. All behavioral evaluations were conducted within the hours of 09:00 to 15:00, within a Controlled environment characterized by low illumination and minimal auditory disturbance. Mice were subjected to a period of environmental acclimatization spanning three days before the initiation of experiments. It's important to note that all behavioral testing and subsequent data analyses were carried out by experimenters who were blinded to the specific treatments administered to the mice. In order to replicate prophylactic treatment, assessments were conducted prior to each NTG injection.

### Hindpaw mechanical threshold

The quantification of hindpaw mechanical thresholds in mice was performed as previously described [45]. Mice were individually placed on a wire rack for 30 min to allow acclimatization. Eight von Frey (Aesthesio®, Danmic Global, San Jose, CA, USA) filaments (0.04–2 g) were applied to the center of the plantar surface of the hind paw at an initial stimulus intensity of 0.16 g. We considered a positive response if we identified shaking, twitching, or paw licking after the procedure. Each filament was applied to the skin for 3 s at least 1 min intervals. Mechanical thresholds were tested and calculated using an upward and downward approach; the right hind paw was stimulated five times, and three or more positive responses out of five stimuli were considered positive responses to that filament. If there was no response to that stimulus, the filament strength increased; otherwise, the filament strength decreased.

### Periorbital mechanical threshold

To quantify head allodynia, the foreheads of the mice were shaved and the mice were acclimatized for at least 3 days. We performed the test by gently placing the mouse in the palm of the hand and applying von Frey filaments vertically to the shaved skin. All the other tests were performed in the same way as for plantar thresholds. Scratching the face with the forepaw and rapid withdrawal were considered positive responses.

### Light-aversive test

Photophobia is a typical accompanying symptom of migraine, so we used a light–dark box (Shanghai Xinruan Information Technology Co., Ltd., XR-XB120, Shanghai, China) to test the photophobic performance of NTG model mice.

The box (30 cm wide × 30 cm deep × 30 cm high) consisted of two compartments of equal size: the light box was painted white and illuminated with LEDs, etc. (≥ 635 lx), while the dark box was painted black and unlit (≤ 5 lx). The channel (7 cm × 7 cm) connected the two compartments and allowed the mice to move freely. Each mouse was gently placed in the middle channel on the day of the test and the time spent in the light box was recorded over a 10-min period using the manufacturer's software. Light intensity settings are from previous studies [39]. To simulate prophylactic treatment, we tested each mice 24 h after the last dose.

### Elevated plus maze (EPM) test

We used an elevated plus maze (EPM; Shanghai Xinruan Information Technology Co., Ltd., XR-XG201, Shanghai, China) to assess the anxiety-like behaviour associated with migraine. The apparatus was placed 60 cm above the floor and consisted of four arms (35 cm wide × 5 cm deep), two of which have 15 cm high walls forming a 'closed' arm, while the 'open' arm acts as an open runway. Mice were placed in the centre of the maze facing one of the open arms and allowed to freely explore the EPM for 5 min. Anxious rodents were more inclined to show greater avoidance of the open arms. Record how long the mice stayed in the open arm and how many times they entered the open arm using the manufacturer's software. To simulate prophylactic treatment, we tested each mice 24 h after the last dose.

### Immunofluorescence staining

The mice were anaesthetized with 1.25% avertin and a transcranial perfusion with 40 ml PBS (pH 7.2–7.4) and 20 ml 4% paraformaldehyde (PFA). Hearts were fixed in PFA (4%) for 24 h at 4 °C and then dehydrated in 15% and 30% sucrose solution. Coronal sections of 30 μm were cut using a freezing microtome (Leica Biosystems, CM1950, Heidelberger, Germany). Sections were permeabilized with 0.25% Triton X-100 (Beyotime, Shanghai, China) for 10 min at room temperature, then blocked with 10% donkey serum (Boster, Wuhan, China) for 2 h at room temperature and incubated overnight at 4 °C with primary antibody diluted in blocking solution. The next day, the secondary antibody was incubated for 2 h, and 4′,6-diamidino-2-phenylindole (DAPI) was incubated for 10 min at 37 °C. The primary antibody was incubated with 50% glycerol. After sealing with 50% glycerol, we observed the sections using a fluorescence microscope (Olympus, Japan). Negative Control sections were stained with PBS instead of primary antibody and no positive signals were identified. The antibodies used are listed in Table 1.

**Table 1:** Antibodies used in Western blotting and immunofluorescence analysis

Antibody	Manufacturer	Catalog number	Host
For Western blotting analysis			
CGRP	CST, USA	14959S	Rabbit
c-Fos	Abccam, UK	ab214672	Rabbit
Nrf2	CST, USA	12721S	Rabbit
HO-1	Abccam, UK	ab189491	Rabbit
NQO-1	Abccam, UK	ab80588	Rabbit
iNOS	Abccam, UK	ab202417	Rabbit
β-Actin	Proteintech, USA	20536–1-AP	Rabbit
GAPDH	Proteintech, USA	60004–1-lg	Mouse
Fibrillarin	Beyotime, China	AF380	Rabbit
Anti-Rabbit lgG (HRP)	Proteintech, USA	RGAM001	Goat
Anti-Mouse lgG (HRP)	Proteintech, USA	RGAR001	Goat
For immunofluorescence staining			
CGRP	Abccam, UK	ab36001	Sheep
c-Fos	Abccam, UK	ab214672	Rabbit
Alexa Fluor 488 Donkey anti-Goat IgG	Abccam, UK	ab150177	Donkey
Alexa Fluor 594 Donkey anti-Rabbit IgG	Abccam, UK	ab150076	Donkey

For CGRP, the fluorescence signal area ratio of the SP5C was analyzed using ImageJ software (Fiji, NIH, USA). For c-Fos, the number of c-Fos positive cells was quantified by taking the same field of view in the superficial layer of the SP5C at 20 × magnification.

### Western blot analysis

Fresh SP5C tissues were subjected to homogenization using a cryomill (Servicebio, Wuhan, China) and radioimmunoprecipitation (RIPA) lysis buffer (Beyotime, Shanghai, China) supplemented with phenylmethylsulfonyl fluoride (PMSF, Shanghai, China). The homogenates were pre-cooled to -30°C, with homogenization cycles of 30 s each (two cycles in total, with a 15-s interval). The protein concentration was quantified utilizing a bicinchoninic acid (BCA) protein analysis kit (YangGuangBio, Beijing, China). Equal amounts of proteins (30 μg per lane) were separated on 4%-20% gradient SDS-PAGE gels (ACE Biotechnology, Shanghai, China) and subsequently transferred to polyvinylidene difluoride (PVDF) membranes (Millipore, USA). The membranes were initially blocked for 1 min at room temperature using 1-min Fast Blocking Buffer for Western (YangGuangBio, Beijing, China). Following this, an overnight incubation at 4°C was performed with the primary antibodies listed in Table 1. The subsequent day, the membranes underwent a series of three 10-min washes with TBST (YangGuangBio, Beijing, China) and were then subjected to incubation with the corresponding peroxidase-linked primary antibodies for 1 h at room temperature. Immunoblotted proteins were visualized utilizing an imaging system (Tanon, Shanghai, China) coupled with a SuperFemto ECL chemiluminescence kit (Vazyme, Nanjing, China). A comprehensive list of the antibodies employed can be found in Table 1.

### ROS detection of SP5C

We took 20 mg of fresh SP5C tissue and added 400 μL of buffer to make a homogenate, which was homogenized using a cryo-mill (servicebio, Wuhan, China) and centrifuged at 4°C for 10 min. 200 μL of supernatant was collected and incubated with 20 μL of BBcellProbeTM O11 ROS probe (BestBio, China) in a 96-well plate at 37°C for 30 min. A fluorescent enzyme marker (Thermo Fisher Scientific, USA) was used to detect ROS levels with an excitation wavelength of 488 nm and an emission wavelength of 530 nm. All samples were repeated 3 times.

### Enzyme‑linked immunosorbent assay (ELISA)

We used specific ELISA kits to detect CGRP in mouse plasma (Sunshine Bio, Beijing, China). Orbital blood is closer to the central nervous system, so we used orbital blood to detect CGRP. Briefly, after the mice were anaesthetized with 1.25% Avertin, the eyeballs were immediately removed and the orbital blood was collected in an anticoagulation tube, immediately centrifuged and processed (4°C, 3000r, 10 min) and then quickly frozen in a -80°C refrigerator as a backup. For the assay, samples were added to the appropriate microenzymatic plate wells and conjugated with specific antibodies according to the instructions. Enzyme reagent was added, the plate was sealed with parafilm and incubated at 37°C for 60 min. After washing the wells with Wash Buffer, we added Colour Developer A and B to each well and incubated the samples for 15 min at 37°C. Finally, we added the termination solution to each well and read the optical density (OD) at 450 nm.

A specific ELISA kit was also used to detect TNF-α, IL-6, IL-1β, SOD and MDA and levels in SP5C (Meike, Shanghai, China). Tissues were weighed and added to PBS (pH 7.2–7.4, 0.01 mol/L) at a ratio of 1:9 and homogenized at -30°C. Samples were centrifuged at 5000 rpm for 15 min and the supernatant was collected. After that, we added the samples to the appropriate microplate wells and conjugated them with specific antibodies according to the instructions. After adding the enzyme reagent, the plate was sealed with a membrane and incubated at 37°C for 60 min. Then, we washed the wells with Wash Buffer, added Chromogen A and B to each well, and incubated the samples for 15 min at 37 °C. Finally, we add Stop Solution to each well and read the optical density (OD) at 450 nm.

### Statistical analysis

All experiments and data analyses were performed by experimenters’ blind to the subgroups. Sample sizes were determined on the basis of previous experience and were all biological replicates. Data are expressed as mean ± standard error of the mean (S.E.M.). GraphPad Prism 9 (GraphPad Software Inc., San Diego, CA, USA) was used for graph generation and statistical analysis. We also relied on an independent samples t-test to assess differences between the two groups. One-way analysis of variance (ANOVA) and Dunnett's multiple comparison test were used for multiple group comparisons. Two-way repeated-measures analysis of variance (ANOVA) was used to analyze behavioural data. Two-tailed tests were used for all statistical analyses, and *p* < 0.05 was considered statistically significant.

### Supplementary Information

**Supplementary Material 1.**

**Supplementary Material 2.**

## Data Availability

No datasets were generated or analysed during the current study.

## Abbreviations

NTG: Nitroglycerin
CM: Chronic migraine
SP5C: Spinal trigeminal nucleus caudalis
NBP: Dl-3-n-butylphthalide
Nrf2: Nucleus factor erythroid 2-related factor 2
iNOS: Inducible Nitric Oxide Synthase
ROS: Reactive Oxygen Species
CGRP: Calcitonin gene-related peptide
PBS: Phosphate-buffered saline
PFA: Paraformaldehyde
PVDF: Polyvinylidene difluoride
DAPI: 40,6-Diamidino-2-phenylindole
WB: Western blotting
IF: Immunofluorescence staining
EPM: Elevated plus maze
