# Supplementary material for: Unveiling the therapeutic potential of Dl-3-n-butylphthalide in NTG-induced migraine mouse: activating the Nrf2 pathway to alleviate oxidative stress and neuroinflammation
Source: J Headache Pain. 2024 Apr 2;25(1):50. doi: 10.1186/s10194-024-01750-1 (PMC10986135; doi:10.1186/s10194-024-01750-1)
Supplement: Supplementary file 1 — Supplementary Material 1. [file 10194_2024_1750_MOESM1_ESM.pdf]

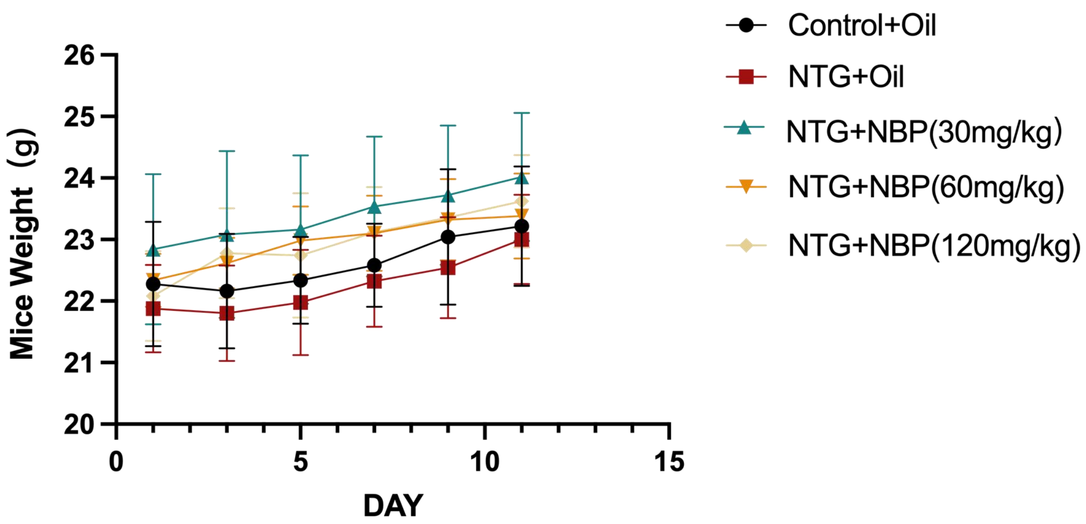

Figure S1. The continuous gavage of NBP did not lead to significant weight reduction in mice.

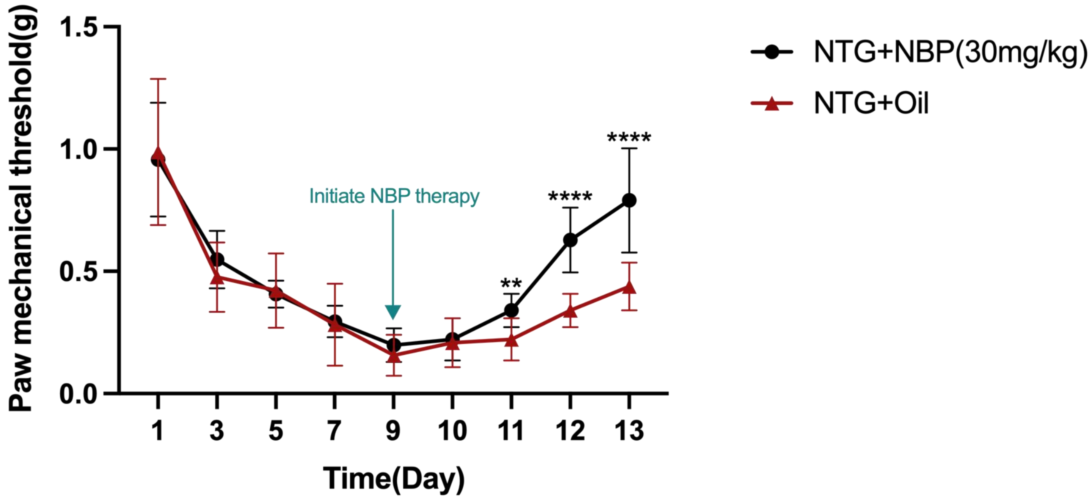

Figure S2. After the completion of the ninth day of modeling, NBP treatment was administered, and the mechanical hyperalgesia in mice gradually recovered. (n=6-8 in each group, **$p < 0.01$ and ****$p < 0.0001$ compared with the DAY9)

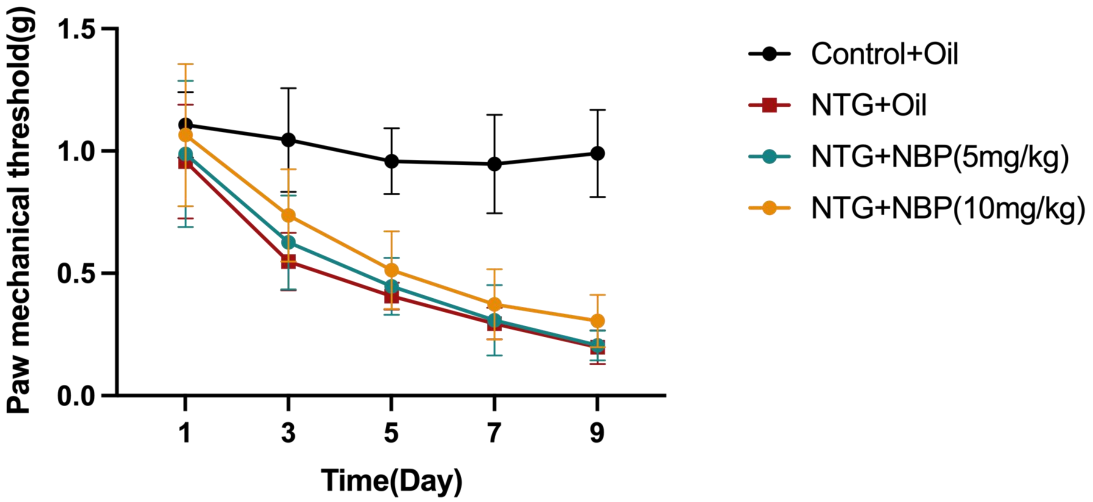

Figure S3. Pre-treatment with a smaller dose of NBP does not improve mechanical pain sensitization in mice. (n=6-8 in each group)
